# Preliminary study on AI-assisted diagnosis of bone remodeling in chronic maxillary sinusitis

**DOI:** 10.1186/s12880-024-01316-2

**Published:** 2024-06-10

**Authors:** Caiyun Zou, Hongbo Ji, Jie Cui, Bo Qian, Yu-Chen Chen, Qingxiang Zhang, Shuangba He, Yang Sui, Yang Bai, Yeming Zhong, Xu Zhang, Ting Ni, Zigang Che

**Affiliations:** 1 Department of Radiology, Nanjing Tongren Hospital, School of Medicine, Southeast University, No. 2007, Ji Yin Avenue, Jiang Ning District, Nanjing, 211102 PR China; 2 Department of Radiology, Nanjing First Hospital, Nanjing Medical University, Nanjing, PR China; 3 Department of Otolaryngology Head and Neck Surgery, Nanjing Tongren Hospital, School of Medicine, Southeast University, Nanjing, PR China; 4 School of Statistics and Management, Shanghai University of Finance and Economics, Shanghai, PR China

**Keywords:** Bone remodeling, Chronic maxillary sinusitis, Computed tomography imaging, Artificial intelligence (AI), Deep learning, Machine learning

## Abstract

**Objective:**

To construct the deep learning convolution neural network (CNN) model and machine learning support vector machine (SVM) model of bone remodeling of chronic maxillary sinusitis (CMS) based on CT image data to improve the accuracy of image diagnosis.

**Methods:**

Maxillary sinus CT data of 1000 samples in 500 patients from January 2018 to December 2021 in our hospital was collected. The first part is the establishment and testing of chronic maxillary sinusitis detection model by 461 images. The second part is the establishment and testing of the detection model of chronic maxillary sinusitis with bone remodeling by 802 images. The sensitivity, specificity and accuracy and area under the curve (AUC) value of the test set were recorded, respectively.

**Results:**

Preliminary application results of CT based AI in the diagnosis of chronic maxillary sinusitis and bone remodeling. The sensitivity, specificity and accuracy of the test set of 93 samples of CMS, were 0.9796, 0.8636 and 0.9247, respectively. Simultaneously, the value of AUC was 0.94. And the sensitivity, specificity and accuracy of the test set of 161 samples of CMS with bone remodeling were 0.7353, 0.9685 and 0.9193, respectively. Simultaneously, the value of AUC was 0.89.

**Conclusion:**

It is feasible to use artificial intelligence research methods such as deep learning and machine learning to automatically identify CMS and bone remodeling in MSCT images of paranasal sinuses, which is helpful to standardize imaging diagnosis and meet the needs of clinical application.

## Introduction

Chronic rhinosinusitis (CRS) is a kind of chronic inflammatory disease of the nasal-sinonasal mucosa [1] with a high incidence and recurrence rate [2–4]. An important reason for the development of this condition is the long-term stimulation of inflammatory mucosal lesions, which cause changes in the submucosal sinus wall bone and lead to persistent inflammatory disease. Its pathogenesis is very complex [5–7] and has not yet been clarified [8, 9]. Related pathological studies have confirmed that bone changes in the sinus wall are caused by inflammation, osteogenesis, osteotomy and new bone remodeling [10]. During bone remodeling of the deep maxillary sinus [11], the vast majority of nasal endoscopies have difficulty obtaining pathological samples, while computed tomography (CT) can show changes in bone thickness and density and mucosal thickening [12, 13], common methods for evaluating the presence of chronic maxillary sinusitis (CMS) and CMS with bone remodeling. The assessment of CRS with osteitis encompasses both integral and local factors. At present, the evaluation of CRS by MSCT mainly focuses on the bone thickness and CT attenuation (Hounsfield unit, HU). In the past, bony changes in CRS were considered to be positively correlated with bone thickness and attenuation [14, 15]. Recent studies have demonstrated that CT scans can comprehensively assess the location, extent of involvement, computed tomography attenuation, and bone thickness in chronic maxillary sinusitis (CMS) with bone remodeling [16]. This radiological evidence of bony changes provides a thorough understanding of CRS propagation, offering clinicians valuable information to establish a foundation for uncovering future pathogenic insights.

Artificial intelligence (AI) methods, such as machine learning, artificial neural network (ANN) and convolutional neural network (CNN) [17–19], are of great value in the segmentation, reconstruction, identification, and classification of the data from CT, MRI and other imaging modalities [20, 21]. CNN leverages multi-layer convolutions to excel in various end-to-end medical diagnostic tasks by capturing intricate features in images [19, 22]. Despite their efficacy, the intricate network design comes with drawbacks such as increased computational demand, model complexity, and limited interpretability. On the other hand, support vector machine (SVM) is grounded in statistical learning theory, achieving medical classification tasks by determining the hyperplane with the maximum feature space margin. Nevertheless, SVM necessitates manual feature extractor and kernel function design for handling nonlinear data, presenting challenges in user experience [23]. With the rise of deep learning, particularly the successful integration of CNN, SVM might has been surpassed in some medical image processing tasks. However, SVM excel in finding the optimal balance between model complexity and learning ability with limited sample information. In essence, while CNN dominates in certain aspects of medical image processing, SVM can be based on limited sample information and identify the best compromise between model complexity and ability to learn. SVM have great advantages in solving small-sample and high-dimensional pattern recognition problems [24]. Artificial intelligence methods based on CT big data have gradually played an increasingly important role in head and neck diseases in terms of physical examination and screening, preoperative diagnosis, staging, grading, and efficacy and prognosis evaluation [25]. However, there are few reports on the use of deep learning and machine learning in chronic sinusitis and bone remodeling, as most have typically focused on the diagnosis of maxillary sinusitis [26, 27]. Chowdhury et al. [28] used a CNN to automatically classify the ostiomeatal complex (OMC). The open and closed status of the OMC of the patients was detected by using two-dimensional CT images. Humphries [29], based on multicenter research established an automatic segmentation CNN model. By performing 3D analysis of the volume of chronic sinusitis inflammation as a percentage of the sinus cavity volume. A recent paper reports an automated, deep learning-based algorithm for quantitative sinus CT analysis [30]. However, these studies ignored the analysis of bone remodeling in sinusitis.

Therefore, this study intends to use deep learning (in the form of a CNN) and machine learning (in the form of an SVM) to identify the changes in CMS and bone remodeling on CT and to explore their value in the diagnosis of CMS and bone remodeling.

## Materials and methods

This study was approved by the ethics committees of Nanjing Tongren Hospital, School of Medicine, Southeast University; This research did not cause additional adverse reactions or risks to subjects, and therefore exemption from informed consent was requested.

### General information

A total of 500 patients (1000 sides) were enrolled from January 2018 to December 2021. A total of 327 were males with a mean age of 37.32 ± 17.88 years, and 173 were females with a mean age of 42.03 ± 18.27 years. The exclusion criteria included (1) fungal sinusitis; (2) benign and malignant sinus tumors; (3) sinus cysts and cystic fibrosis; (4) jaw and sinus trauma; (5) osteitis; and (6) other nonchronic sinusitis patients with sinus bone changes.

### CT equipment and scanning method

A 256-row spiral CT scanner (GE, Revolution) was used to collect the volumetric CT images of the paranasal sinus with the following scanning protocol: tube voltage 120 KV, tube current 230 mA, field of view (FOV) 17 × 17 cm, layer thickness and spacing 0.625 mm, and matrix 512 × 512. CT scan range and posterior reconstruction algorithm: the baseline was parallel to the line of the Audio-orbital line, ranging from the top of the frontal sinus to the lower edge of the maxillary alveolar process. Following an axial volume CT scan for all patients, cross-sectional, sagittal, and coronal multiplanar reorganization (MPR) was performed on a randomized workstation. Reconstruction parameters: layer thickness 1 mm, 1 mm interval, window position 200 HU (adjustable), window width 2000 HU.

### Technical roadmap

**Fig. 1:**
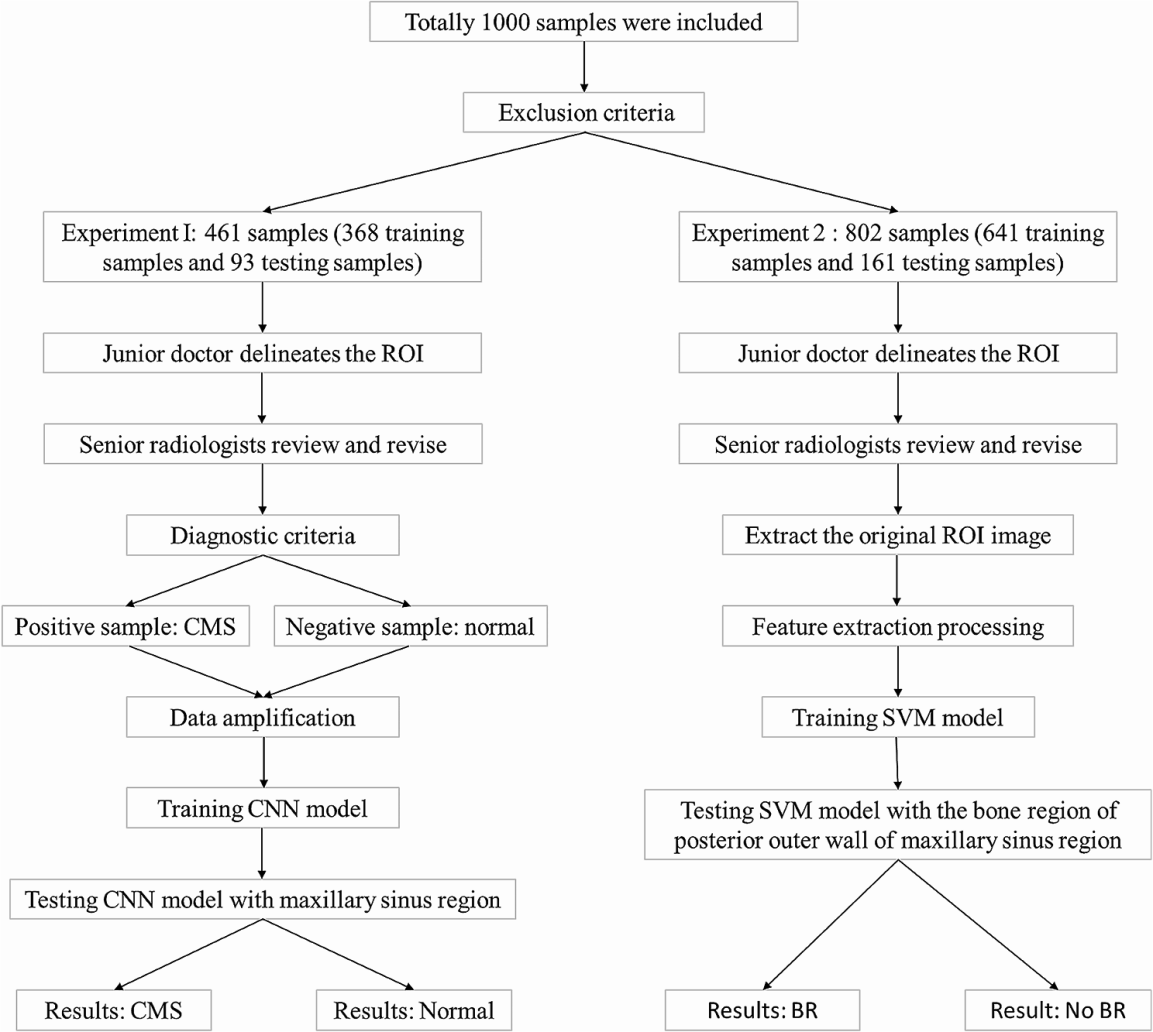
Technical roadmap of experimental steps and methods

The technical roadmap of this study is shown in Fig. 1, involving two experimental approaches. Deep learning and machine learning classification algorithms were used to predict whether the left and right sinuses were from normal or sinusitis patients and bone remodeling with sinusitis. Considering that some patients with sinusitis will have accompanying bone changes, we needed to further predict whether the patients had osteitis. Due to the difficulty in diagnosis and the small size of the sample, we employed a two-stage experimental model: the first stage is based on a deep learning model for performing rapid binary classification for chronic maxillary sinusitis, and the second stage is used to further identify whether chronic maxillary sinusitis patients have bone changes. The combination of the two stages can operate even with seriously imbalanced data, providing a good prediction of sinusitis or even osteitis.

For the first stage, 461 sides were included (368 for the training set and 93 for the test set); 802 sides were included for the second stage (641 for the training set and 161 for the test set).

### Model building and testing

#### Establishment and testing of the model for detecting chronic maxillary sinusitis

**Fig. 2:**
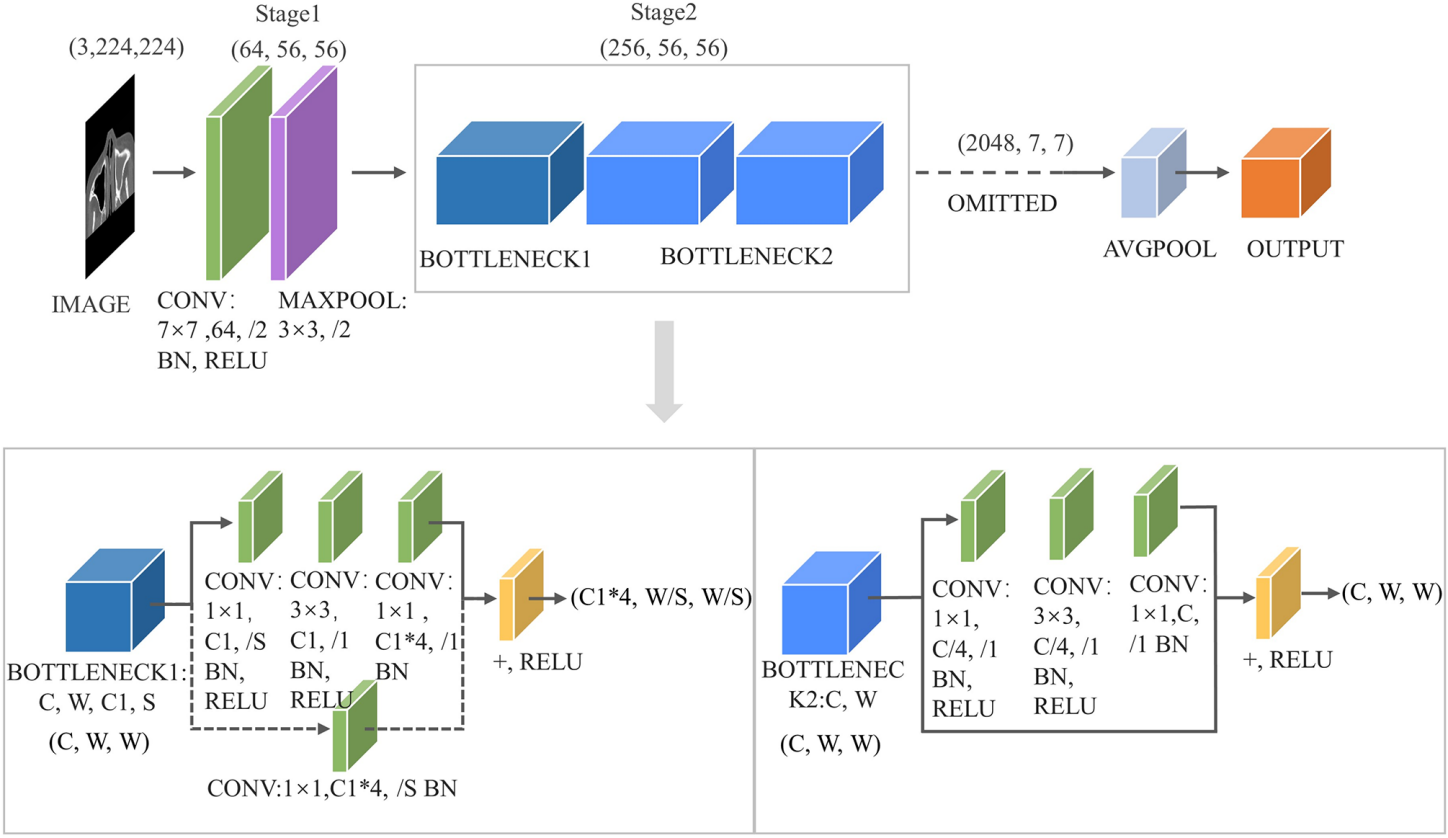
The learning process of CMS by deep learning method based on CNN

**Fig. 3:**
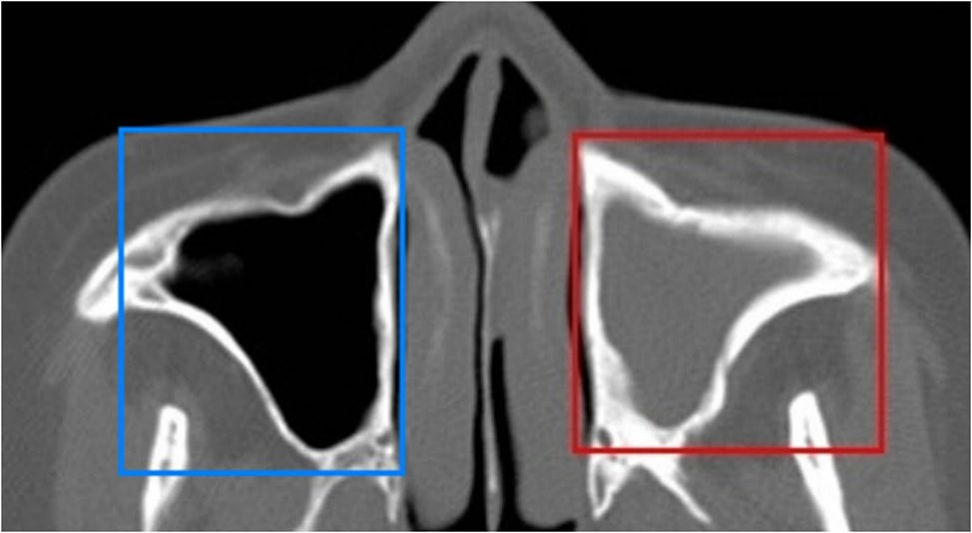
The ROI area of Image annotation delineates (blue box for normal group, red box for pathological group)

The learning process of the CNN-based deep learning method with upper jaw sinus images in DICOM format is shown in Fig. 2. First, the images were imported into the DARWIN intelligent scientific research platform (https://arxiv.org/abs/2009.00908) by a low-seniority doctor. Then, using the annotation system Bbox3D, the doctor outlined the lesions and normal control areas as regions of interest (ROI), as shown in Fig. 3. Two doctors with more than 10 years of experience in nasal imaging diagnosis then reviewed and verified the ROI areas to ensure that they contained the complete maxillary sinus cavity and jointly determined the presence of maxillary sinusitis according to the CMS diagnostic criteria: soft tissue density shadow in the sinus cavity and greater than 3 mm of mucosal thickening. The 461 side images were allocated to the training set and test set at a 4:1 ratio; the CNN model was trained with the training set after image enhancement. The 93 side-image test set was then applied to the optimal model for testing, and the results were recorded.

#### Establishment and testing of the model for detecting bone remodeling in chronic maxillary sinusitis

**Fig. 4:**
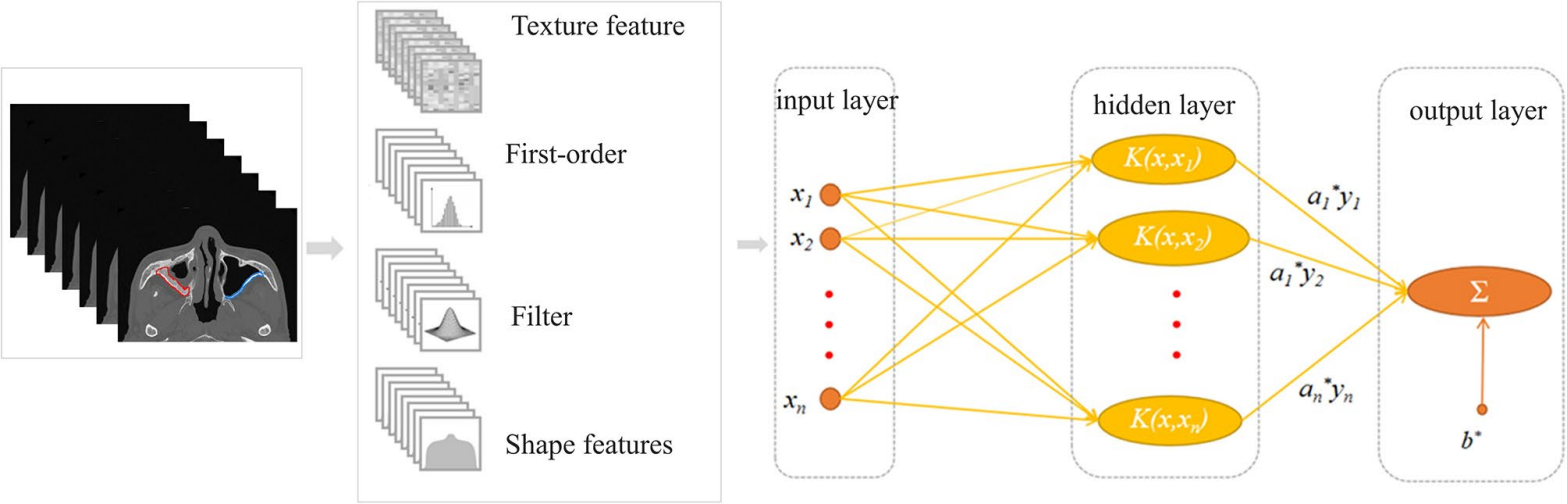
The learning process of CMS with bone remodeling by machine learning method based on SVM

**Fig. 5:**
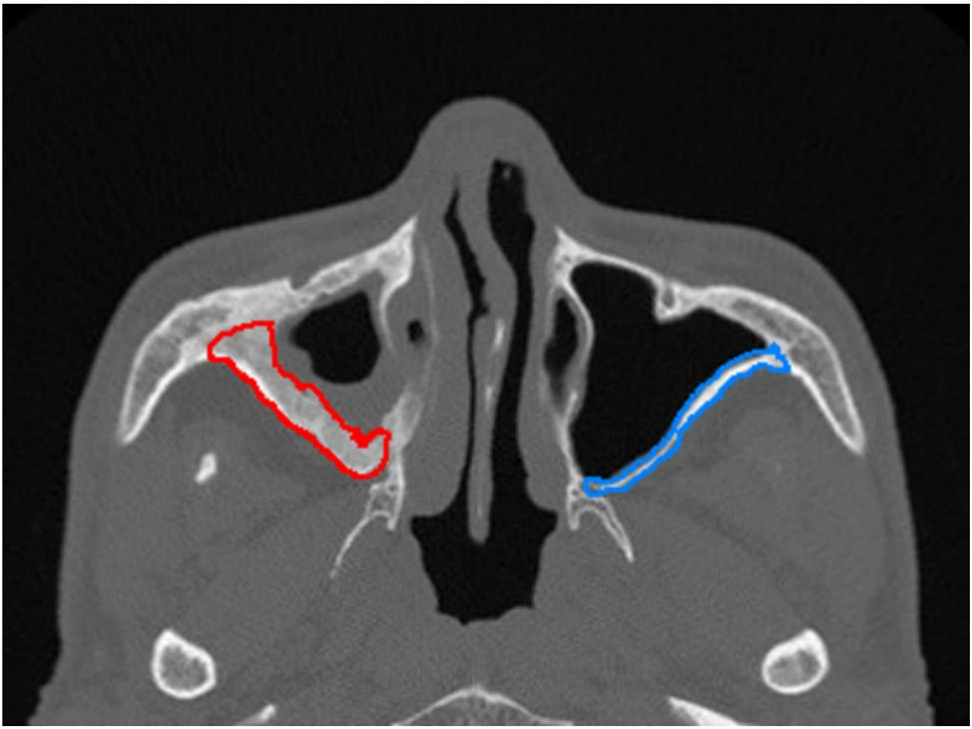
the ROI area of Image annotation delineates (blue for normal group, red for pathological group)

The learning process of the SVM-based model using the DICOM-format maxillary sinus images is shown in Fig. 4. First, the images were imported into the DARWIN intelligent research platform (https://axis.org/abs/2009.00908). One low-seniority doctor and two high-seniority radiologists with more than 10 years specializing in nasal imaging diagnosis determined the measurement site for chronic maxillary sinusitis [31] and separately delineated the ROI along the edge of the lesion in Bbox3D using a thin bone window, as shown in Fig. 5. The diagnostic criteria of bone remodeling in chronic maxillary sinusitis were based on the GOSS score and the improved GOSS score. According to the CT evaluation of sinusitis bone remodeling in the previous stage [16], the thickness and accumulation range of maxillary sinusitis were measured to determine whether sinusitis bone remodeling occurred. Specifically, if the thickness of the sinus wall exceeded 3 mm, it was indicative of sinusitis bone remodeling. The 802 images designated for this model were assigned to a training set and test set at a ratio of 4:1. After min-max normalization, the SVM model was trained according to the model requirements. We generated a Receiver Operating Characteristic (ROC) curve based on the weights assigned to different features in the model. The validity of the established model was confirmed using a 5-fold cross-validation method. The 161 side-image test set was applied to the optimal model for testing, and the output was recorded.

### Statistical analysis

Statistical analysis was performed using SPSS 21.0 software. Numerical data are expressed as the mean and standard deviation (X ± s) or median and quartiles. The optimal model index was recorded by the DARWIN platform, and then the receiver operating characteristic (ROC) curve was drawn according to the weighted contributions of the different characteristics of the model. The model was tested on the test set to draw the ROC curves and determine the performance in terms of the sensitivity, recall, specificity, positive predictive value (PPV), negative predictive value (NPV), F1 score, accuracy and area under the curve (AUC) values.

$$\begin{gathered} acc=\frac{{TP+TN}}{{TP+FN+FP+TN}} \hfill \\ sens=\frac{{TP}}{{TP+FN}} \hfill \\ spec=\frac{{TN}}{{TN+FP}} \hfill \\ ppv=\frac{{TP}}{{TP+FP}} \hfill \\ npv=\frac{{TN}}{{TN+FN}} \hfill \\ {F_1}=\frac{{2TP}}{{2TP+FP+FN}} \hfill \\ \end{gathered}$$

TP True Positive; TN True Negatie; FN False Negative; FP False Pocitive; acc (accuracy); sens (sensitivity); spec (specificity).

## Results

### Model establishment and test results in detecting CMS

**Fig. 6:**
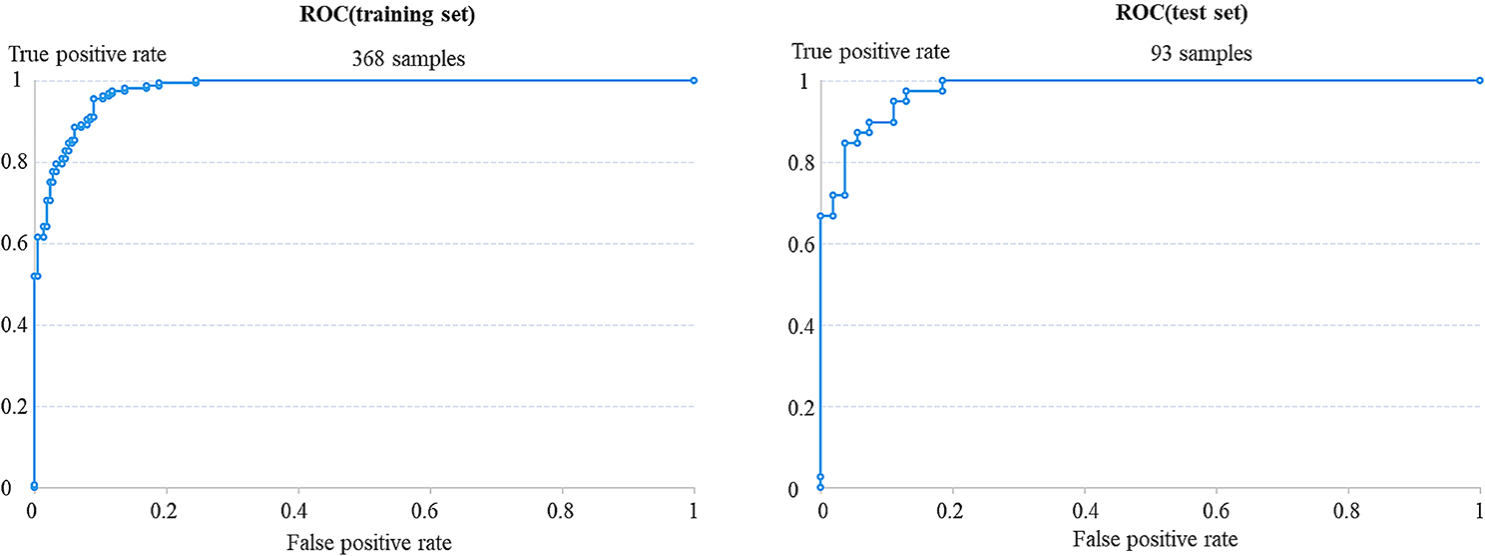
The ROC curve of CNN model on training set and test set

The optimal threshold was determined according to Youden’s index, and the model plateau suggested that it performed best by epoch 90. The ROC curves corresponding to the training and test set are shown in Fig. 6, yielding AUC values of 0.98 and 0.94, respectively. The performance metrics of this model in the test set are shown in Table 1.

**Table 1:** CNN model performance in test set of detecting chronic maxillary sinusitis

Evaluation index	Value	95%CI
Sensitivity	0.9796	0.8931,0.9964
Specificity	0.8636	0.7329,0.9360
PPV/Precison	0.8889	0.7781,0.9481
NPV	0.9744	0.8682,0.9955
F1 Score	0.9320	/
Accuracy	0.9247	/

### Model establishment and test results in detecting bone remodeling in CMS

**Fig. 7:**
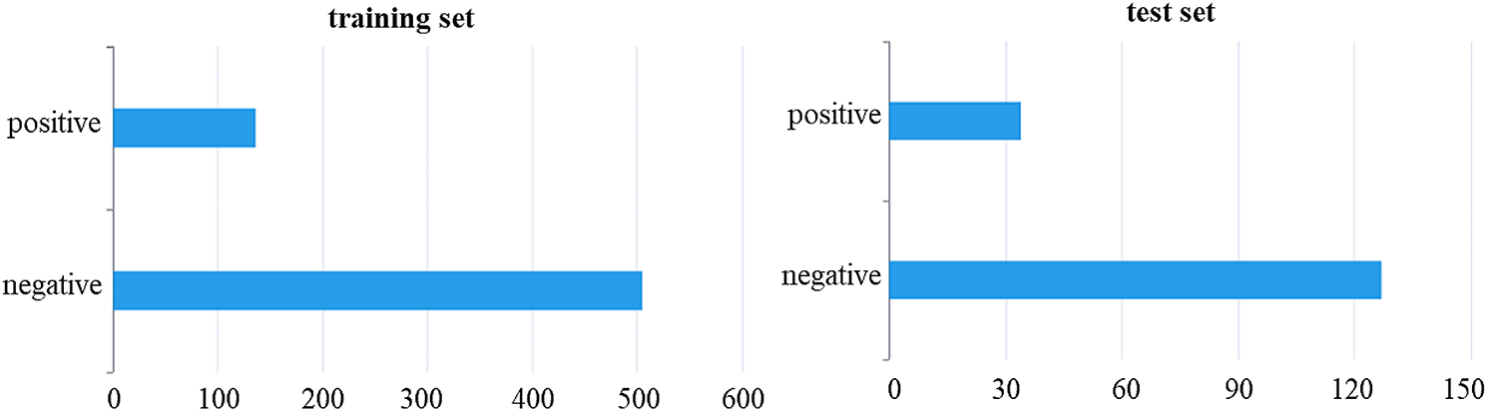
Data distribution of samples in the training set and test set

**Fig. 8:**
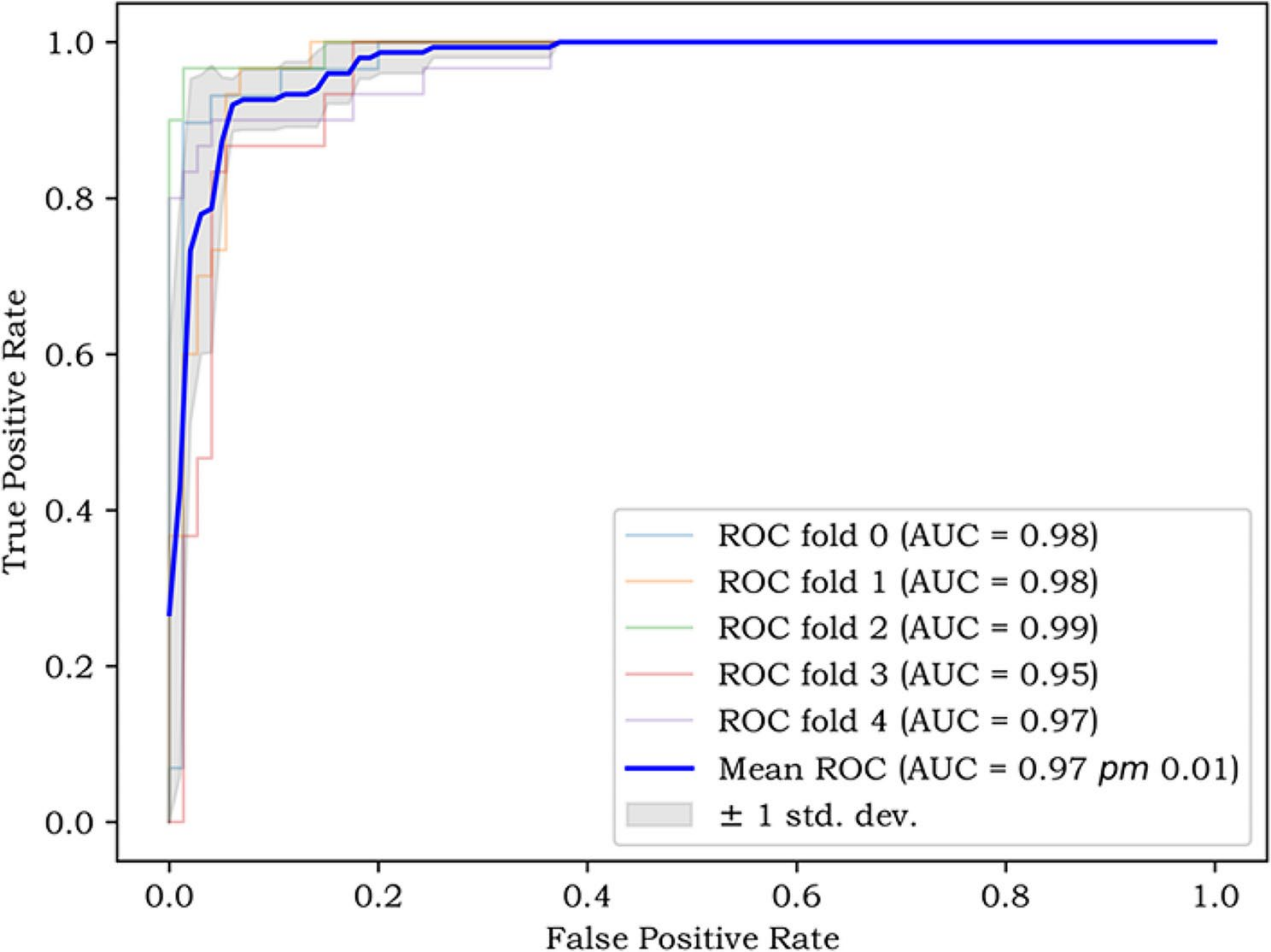
The 5-fold cross-validation of SVM model

**Fig. 9:**
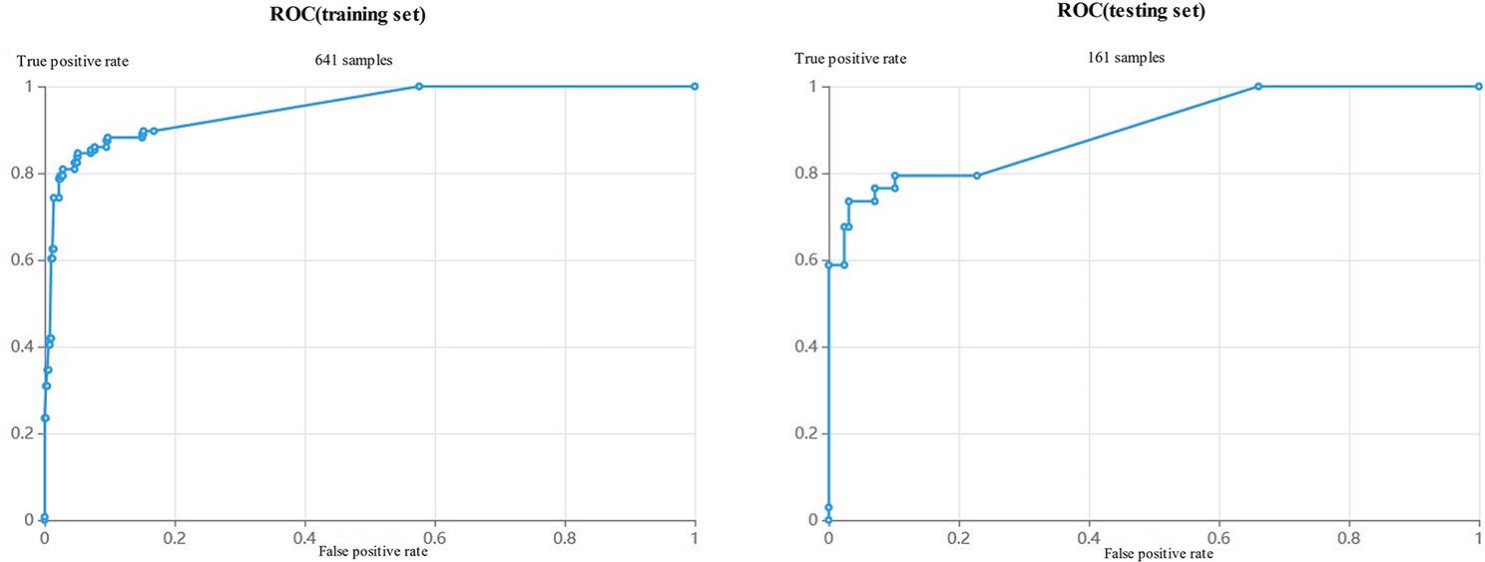
ROC curve of SVM optimal model and ROC curve of test set

A total of 641 side images were used to build the model, and 161 side images were used exclusively to verify the model. The distribution of samples in the training set and test set is shown in Fig. 7. The optimal model index was recorded using the DARWIN platform, and the 5-fold cross-validation results of the model are shown in Fig. 8. The ROC curves of the training and test sets were drawn according to the weighted contributions of different features to the model, as shown in Fig. 9. The AUC values of the training and test sets were 0.94 and 0.89, respectively. The performance metrics of the model in the test set are shown in Table 2.

**Table 2:** SVM model performance in test set of detecting bone remodeling in chronic maxillary sinusitis

Evaluation index	Value	95%CI
Sensitivity	0.7353	0.5688,0.8540
Specificity	0.9685	0.9218,0.9877
PPV/Precison	0.8621	0.6944,0.9450
NPV	0.9318	0.8755,0.9637
F1 Score	0.7937	/
Accuracy	0.9193	/

## Discussion

Chronic sinusitis is a chronic inflammatory disease of the sinonasal mucosa, usually characterized by mucopurulent mucus, intermittent or persistent nasal congestion, nasogenic cephalofacial pain and/or loss of hyposmia, with symptoms recurrent or lasting for more than 3 months. Chronic sinusitis has a long course, easily relapses, is highly heterogeneous, and presents with diverse clinical manifestations and a complex etiology; consequently, there is a lack of early, direct and effective diagnostic methods for the disease and of accurate and objective assessments of the severity of the disease and its prognosis after treatment [32, 33]. Chronic sinusitis bone remodeling causes intractable sinusitis, leading to delayed treatment and imposing a heavy burden to the patients. Thus, this disease has attracted increasing attention from researchers.

CT is the preferred modality for the noninvasive evaluation of the nasal sinuses, alongside clinical symptoms assessment and nasal endoscopy, and plays a key role in the evaluation of chronic sinusitis and bone remodeling. For example, the Lund-Mackay scoring system [34, 35] provides a simple and practical semiquantitative visual scoring technique for sinus CT that is associated with clinical measures of disease severity; however, this system is inconsistent with regard to disease symptoms, time-consuming, and heavily subjective, it is insensitive to changes, and it cannot reflect the inflammation of each sinus [36, 37]. As the demand for healthcare increases, the sinus CT examination workflow, including the time required for a diagnosis and the doctors’ relevant workload, is increasing; therefore, there is increasing demand for a quick, efficient, and accurate method for diagnosing chronic sinusitis and bone remodeling, and the establishment of a prediction model for this condition that can serve as an auxiliary tool for physicians would have important clinical implications. In recent years, some scholars have begun researching ways to develop such methods. Such as sinonasal virtual 3D modelling, airflow modelling and 3D printing for clinical experiments. Their research provides evidence that model analysis can effectively simulate the post-sinus operation changes in mucous membrane, nasal airflow, and environmental conditions. The use of 3D printing emerges as a rapid and efficient means to replicate nasal structures, facilitating experimental tests to evaluate nasal function. This underscores the significance of 3D printing as a valuable auxiliary tool for conducting nasal pressure measurements [38, 39]. Computer methods for quantifying the sinus cavity contents on CT have shown potential, but existing methods rely on manual or semiautomatic segmentation of the sinus cavity, which is also time consuming and unsuitable for routine clinical use. Therefore, a fully automated system for CT sinus segmentation can help to efficiently and objectively quantify nasal sinus inflammation with clinical utility as a screening tool.

This study involves two AI methods, a convolutional neural network and a support vector machine. Due to the high incidence of CMS, the difficulty in identifying the disease and the lack of a sufficient sample size, the recognition and diagnosis of normal or CMS images can be achieved by combining two deep learning classification algorithms. Therefore, the goal of this study is the development of a rapid recognition model for CMS based on the CNN deep learning method. An important reason for the refractoriness and recurrence in CMS is sinus wall bone remodeling; however, identification of this condition is also difficult, as it requires a high degree of specialization to locate easily missed signs. Therefore, we performed a second experiment to attempt to establish an AI prediction model for bone remodeling in CMS. Considering the variable difficulty in the diagnostic identification of bone remodeling and the uneven distribution of the data, experiment 2 of this study adopted the SVM machine learning model, which can achieve stable performance even with a small sample. The combination of these two methods can aid in predicting CMS and bone remodeling in CMS even with unbalanced data amount.

### Deep learning AI-assisted diagnosis of CMS based on CT data

In this paper, a deep learning CNN model was established and tested based on the CT data normal and inflamed sinuses. The AUC value of the training set reached 0.98 and was relatively stable. In the test set (consisting of the images from 93 sides), the sensitivity, specificity, PPV, NPV, F1 score and accuracy of the CMS detection model were 0.9796, 0.8636, 0.8889, 0.9744, 0.9320, and 0.9247, respectively, and the AUC value was 0.94.

In the past, the Lund-Mackay score was used to evaluate the extent and severity of the disease, including the bilateral maxillary sinus, the anterior ethmoidal sinus, and the maxillary sinus which has a relatively fixed anatomy and small variation. However, there are few AI studies on inflammatory sinuses. Berger et al. [40] used an AI platform combined with pathological sections and histological analysis to subclassify cases of CMS. There are also few studies of sinus disease based on imaging data. Murata et al. [26] classified maxillary sinusitis using a CNN based on panoramic X-ray images, achieving an AUC value of 0.87 and an accuracy of 89.6%. Kuwana et al. [27] also used a deep learning method to detect and classify maxillary sinusitis on the basis of panoramic X-line images, with an accuracy rate of 90%. However, these studies used X-ray images; X-ray examinations have low sensitivity and poor resolution for CMS and have been replaced by CT examinations in China. In this study, CT data were applied for AI prediction, which avoided the influence of the overlap and artifacts typical of X-ray images, with an AUC value of 0.94 and good efficiency. In an AI study involving sinus CT data, Chowdhury et al. [28] used a CNN to automatically classify the ostiomeatal complex (OMC). The open and closed status of the OMC of the patients was detected by using two-dimensional CT images. The experimental results showed an accuracy of 85%, and the AUC value in the test group was 0.87. However, the model only considered the effect of a single anatomical factor on CMS and was based only on 2D coronal CT images, while in this experiment, the model was based on 3D images. Consequently, judgement for the entire maxillary sinus cavity was more objective and closer to the ground truth, with an AUC value of 0.94. The result with the test set was also excellent. Humphries [29], based on multicenter research and the sinus CT data of 690 patients, established an automatic segmentation CNN model. By performing 3D analysis of the volume of chronic sinusitis inflammation as a percentage of the sinus cavity volume, the authors evaluated the severity of the sinusitis and provided an objective and quantifiable tool for qualitative sinus disease. This experiment did not seek to classify the severity of CMS, but we expect to undertake further study in this direction in the future.

In this experiment, 9 prediction results were different from the ground truth. The reason for this may be the subjective error in measuring the mucosal thickening in the diagnosis of maxillary sinusitis using CT data. Because the 3 mm boundary was defined as with or without inflammation, there is the possibility of mismeasurement during the measurement, which may have produced bias in the training of the AI model. This experiment focused on the diagnosis of CMS, which achieved good results. We will consider further diagnosing sinusitis in other groups. The second aspect of this study involved establishing a foundation for the AI diagnosis of bone remodeling in CMS.

### Machine learning AI-assisted identification of bone remodeling in CMS based on CT data

The second experiment of this paper involved the exploration of an SVM based on CT data for identifying bone remodeling. Imaging omics were combined with an SVM machine learning model, and the thresholded semiautomatic segmentation method was used to segment the bone area and extract the imaging omics features. Through feature standardization and screening processes, the optimal feature was found, and the bone remodeling discrimination model was established. The training set AUC value was 0.94. In the test set (consisting of 161 side images), the ROC curve indicated better performance, and the AUC value was 0.89. The sensitivity, specificity, PPV, NPV, F1 score and accuracy were 0.7353, 0.9685, 0.8621, 0.9318, 0.7937 and 0.9193, respectively. According to previous studies employing visual assessment [8, 34], the site of bone remodeling in CMS is after the outer wall, which is also an important area for measuring bone remodeling. However, considering the large sample size in the process of the experiment and the low efficiency in outlining the large ROI during the labelling process, this experiment chose CT images of the maxillary sinus posterolateral wall for the area of interest and measured the thickness, density, and abnormal changes in the outline of the structure of the bone remodeling samples.

Previous research on bone-based AI algorithms has focused on [41, 42] bone mineral density and fracture deep learning and prediction. For instance, using data on fractured trunks and limbs, Olczak Scholars et al. [43] used five open deep learning networks to analyze fracture images at different sites. The fracture diagnosis accuracy of the optimal network was 83%. Seok Won Chung et al. [44] evaluated the ability of AI to detect and classify proximal humeral fractures. Using a CNN, the classification accuracy for normal shoulder and proximal humerus fractures was 96%. For different categories of fractures, the model had a classification accuracy of 65 – 86%. Few AI studies have targeted bone changes in the sinus region. However, AI studies of sinus bone remodeling have not been reported thus far.

In this experiment, the machine learning SVM model had high efficiency in detecting bone remodeling in CMS. The ROC curves of the training set and test set were close to the upper left corner, and the AUC value of the test set was 0.89, indicating that the algorithm produced little error in the training process and has high prediction accuracy for bone remodeling with unknown data. However, due to the relatively small sample size and the high degree of fitting in the training process, overestimation of the risk of disease in the test set was very likely. In future experiments, the sample size will be increased to avoid the risk of overfitting. Bone remodeling in CMS involves a wide range of the sinus, although the most common site is the maxillary sinus [45], but it is nevertheless worth further evaluating the other sinus areas, especially the sphenoid sinus if the surgery involves different sinus walls, according to the surrounding anatomy, such as the adjacent blood vessels and nerves. Bone remodeling in these other sinus areas can also show different clinical symptoms, another reason to further expand the sample size and continue to perform systematic research. Based on the good results obtained with the SVM model in the test set, this study offers an effective way to use images and clinical information to obtain an efficient, standardized, and systematic diagnosis of bone remodeling in CMS.

This study also has some limitations. First, the CNN and SVM models may not be fully generalizable due to the limited sample size and insufficient quality optimization; we will continue to accumulate samples in future studies. Second, this study adopts the step-by-step deep learning and traditional machine learning modeling method. Despite the difficulty in obtaining the different diagnoses and the different accuracies, the SVM model has high precision and good stability even with small sample sizes. Relevant experiments will be performed and integrated in future studies.

In summary, this study preliminarily established an auxiliary image evaluation AI model for diagnosing CMS and bone remodeling, which demonstrated desirable test results. On the basis of the gradual healing of the sinus cavity, a systematic evaluation of the sinus wall can not only help standardize the imaging diagnosis of bone remodeling in chronic sinusitis but also improve the efficiency of imaging doctors and provide an additional reference for clinical diagnosis and treatment.

## Data Availability

The datasets used and/or analyzed during the current study are available from. the corresponding author upon reasonable request.

## Abbreviations

CRS: Chronic rhinosinusitis
CT: Computed tomography
CMS: Chronic maxillary sinusitis
AI: Artificial intelligence
CNN: Convolutional neural network
SVM: Support vector machine
ROI: Regions of interest
ROC: Receiver operating characteristic
AUC: Area under the curve
